# Impact of the COVID-19 pandemic on the incidence and mortality of Takotsubo syndrome in Sweden, a report from the SWEDEHEART registry

**DOI:** 10.1186/s12872-026-06889-0

**Published:** 2026-10-29

**Authors:** T. Gudmundsson, P. Petursson, T. Råmunddal, O. Angeras, A. Rawshani, O. Frobert, J. Alfredsson, C. Ekenbäck, L. Henareh, K. Skoglund, C. Ljungman, E. Bollano, M. Mohammad, H. Hagström, T. Jernberg, D. Erlinge, B. Redfors, E. Omerovic

**Affiliations:** 1 Department of Cardiology, Sahlgrenska University Hospital, Gothenburg, Sweden; 2 Department of Molecular and Clinical Medicine, Institute of Medicine, Sahlgrenska Academy, University of Gothenburg, Gothenburg, Sweden; 3 Department of Clinical Medicine, Faculty of Health, Aarhus University, Aarhus, Denmark; 4 Department of Cardiology, Örebro University Hospital, Örebro, Sweden; 5 Department of Health, Medicine and Caring Sciences, Department of Cardiology, Linköping University, Linköping, Sweden; 6 Department of Clinical Sciences, Division of Cardiovascular Medicine, Karolinska Institutet, Danderyd University Hospital, Stockholm, Sweden; 7 Department of Medicine, Heart and Vascular Theme, Karolinska University Hospital, Karolinska Institutet, Stockholm, Sweden; 8 Department of Cardiology, Clinical Sciences, Lund University, Skåne University Hospital, Lund, Sweden; 9 Department of Cardiology, Umeå University Hospital, Umeå, Sweden

**Keywords:** COVID-19, Takotsubo syndrome, Pandemic, Mortality rate, Swedish coronary angiography and angioplasty registry, Acute heart failure

## Abstract

**Introduction:**

Takotsubo syndrome (TTS) is often triggered by intense emotional or physical stress, with symptoms closely resembling those of acute myocardial infarction and heart failure. The COVID-19 pandemic caused unprecedented psychological, social, and economic stress, with TTS incidence and mortality either increasing or remaining stable. Previous studies have relied on single- or multicenter data, lacking the scope to provide national-level insights. This study aimed to fill the gap by investigating the incidence and outcomes of TTS across Sweden during two distinct periods: the pre-pandemic period (January 2015 to March 2020) and the pandemic period (April 2020 to December 2022).

**Methods:**

Using the nationwide Swedish Coronary Angiography and Angioplasty Registry, we assessed the incidence rate (IR) for all patients with TTS referred for coronary angiography in Sweden. We calculated incidence rate ratios (IRRs) both before and during the pandemic using quasi-Poisson regression, adjusted for age and sex. We evaluated 30-day and 1-year mortality using multivariable Cox proportional hazards regression, accounting for clustering within hospitals.

**Results:**

We identified 3,180 patients hospitalized with TTS during the study period: 69% (2,189) pre-pandemic and 31% (991) during the pandemic. The incidence of TTS was 10% lower during the pandemic compared to the pre-pandemic period (IRR 0.90, 95% CI 0.84–0.97, *P* = 0.008). The cohort’s crude all-cause mortality rate was 2.73% at 30 days and 6.10% at 1 year, with no significant difference at 30 days (adjusted HR 1.12, 95% CI 0.69–1.78, *P* = 0.642) or one year (adjusted HR 1.05, 95% CI 0.75–1.46, *P* = 0.780). Compared with studies in other countries where incidence increased or remained unchanged, COVID-19 incidence was similar across the matching periods.

**Conclusions:**

This nationwide observational study found a lower incidence of TTS during the pandemic compared to the pre-pandemic period, while mortality rates remained unchanged. The reduced incidence of TTS in Sweden may reflect the country’s comparatively milder pandemic control measures and more robust social security infrastructure relative to other nations.

**Clinical trial number:**

Not applicable.

**Supplementary information:**

The online version contains supplementary material available at https://doi.org/10.1186/s12872-026-06889-0.

**KEY LEARNING POINTS**.

**What is already known**.

- Studies from multiple countries have shown either increased or stable incidence of Takotsubo syndrome during the pandemic compared to the pre-pandemic period.

**What this study adds**.

- Previous studies were conducted as a single-center trial or had small sample sizes.
- The conflicting results underscore the importance of a large-scale, nationwide registry study to better understand the pandemic’s impact on TTS incidence and outcomes.
- By using a comprehensive national registry offering valuable insights on the effect of the COVID-19 pandemic on TTS incidence for an entire nation.

## Introduction

Takotsubo syndrome (TTS) is often triggered by intense emotional or physical stress, with symptoms closely resembling those of acute myocardial infarction and heart failure [1–3]. In recent years, there has been an increase in TTS cases, potentially due to heightened awareness and improved diagnostic techniques [4, 5]. Reports of increased TTS incidence following major stress-inducing events, such as natural disasters [6], suggest a strong link between stress and TTS onset.

The COVID-19 pandemic caused severe psychological, social, and economic stress [7–12]. Countries implemented different anti-pandemic strategies, significantly impacting citizens’ psychosocial health and stress levels [13]. This period of collective anxiety provided a unique backdrop for assessing the effects on various health conditions, including TTS. Several studies reported increased TTS incidence during the pandemic [14–16], even among patients without active COVID-19 infection, suggesting that social and physical stressors were significant contributing factors. Conversely, other studies [17, 18] found no significant change in TTS incidence during the pandemic, highlighting the need for more comprehensive investigations. Previous studies on TTS during the pandemic had small sample sizes and lacked nationwide data, which limited the broader applicability of their findings.

Both short- and long-term mortality in TTS patients are comparable to those of patients with acute myocardial infarction [4]. During the COVID-19 period, studies have reported either increased or stable mortality rates among TTS patients compared to pre-pandemic periods [14, 17].

The conflicting results underscore the importance of a large-scale, nationwide registry study to better understand the pandemic’s impact on TTS incidence and outcomes.

Our study aimed to investigate the incidence of TTS in Sweden during the pre-pandemic and pandemic periods and to compare mortality rates between these two timeframes. By leveraging data from a comprehensive national registry, we offer valuable insights into the pandemic’s impact on TTS incidence, severity, and outcomes, enhancing our understanding of how external stressors influence this condition. Additionally, to better understand differences in TTS incidence across studies, we sought to compare the incidence of COVID-19 in Sweden with that of other countries that have reported TTS.

## Methods

This observational cohort study uses the SWEDEHEART registry, mainly data from the sub-registry Swedish Coronary Angiography and Angioplasty Registry (SCAAR) [19, 20].

SCAAR records consecutive patients from all 31 hospitals in Sweden that perform coronary angiography and percutaneous coronary interventions (PCI). SCAAR includes 50 variables from all coronary angiographies and approximately 200 from PCI procedures. Patients’ vital status information is updated regularly from the national population registry. The SWEDEHEART registry provides nationwide, high-quality data with nearly complete coverage of patients hospitalized for ACS and undergoing coronary procedures in Sweden. Data completeness for core clinical and procedural variables exceeds 95%, and long-term mortality follow-up is virtually complete through linkage to national population registers.

### COVID-19 data

COVID-19 case and mortality data were obtained from the publicly available dataset provided by Our World in Data (https://ourworldindata.org/covid-cases) [21]. This dataset includes cumulative confirmed cases and deaths per million people across various countries and regions, enabling analysis of pandemic trends over time. For this study, we focused on data from Austria, France, Greece, Italy, Sweden, and the United States, as these countries reported on TTS incidence before and during the pandemic. Data was accessed and analyzed to provide context for studying TTS trends during the pandemic.

### Study population and design

We included patients who underwent coronary angiography and were diagnosed with TTS (*n* = 3180) from January 2015 to December 2022. The interventional cardiologist registers TTS in SCAAR based on the criteria described in Table 1. This TTS criterion has previously been defined and is similar to the current European Society of Cardiology criteria [22, 23]. According to these criteria, other conditions, such as myocarditis or myocardial infarction without concomitant TTS, are excluded. The TTS variable was previously validated in a Swedish cohort from 2005 to 2013, with approximately 9% of suspected TTS cases not meeting the prespecified criteria [4].

**Table 1:** Definition of takotsubo syndrome used in the swedish coronary angiography and angioplasty registry

1. Transient wall motion abnormalities in the left or right ventricle (preceding negative emotional stress or somatic disease, trauma, etc. is often, but not always, present
2. Absence of angiographic signs of plaque rupture (note that significant coronary artery disease may coexist with takotsubo syndrome)
3. New ECG changes (ST-elevation or ST depression or T-wave inversion)
4. Absence of myocarditis.

### Outcome measures

We divided the study period into two periods: pre-pandemic (January 2015 – March 2020) and pandemic (April 2020 – December 2022). The World Health Organization (WHO) classified COVID-19 as a threat to human health on January 30, 2020, and declared it a pandemic in March 2020 [24]. Sweden decided to lift all restrictions in February 2022, and on April 1, 2022, COVID-19 was no longer classified as a threat to public health or a danger to society [25]. However, confirmed COVID-19 cases continued to rise until the end of 2022, then plateaued thereafter [21]. Therefore, we defined the pandemic period from April 2020 to December 2022. We assessed the incidence rate (IR) of TTS across both periods. From the SCAAR registry, we collected data on 30-day and 1-year mortality. Data on mortality was missing for 88 individuals; 54 had foreign Social Security numbers, and information on 34 patients was not updated in the national population registry to SCAAR. Therefore, 3092 patients were included in the survival analyses.

### Statistical analysis

Continuous variables were reported as medians with interquartile ranges (IQRs), and categorical variables as counts and percentages. Comparisons between groups were performed using the Wilcoxon rank-sum test for continuous variables and Pearson’s χ² test for categorical variables. The frequency of TTS was calculated as the number of patients registered with TTS divided by the total number of coronary angiography cases in each study period. All eligible coronary angiography cases were retained in the primary analysis. The association between the pandemic period and TTS diagnosis was evaluated using quasi-Poisson regression with a log link, with TTS as the dependent variable and pandemic period as the independent variable, adjusted for age and sex. Exponentiated regression coefficients were interpreted as relative frequency ratios comparing the pandemic period with the pre-pandemic period. A quasi-Poisson model was used to allow for potential extra-Poisson variation.

Kaplan-Meier estimates were used to calculate cumulative all-cause mortality at 30 days and 1 year among patients with TTS. Mortality was evaluated using Cox proportional hazards regression, with hospital-level clustering accounted for by including a shared frailty term. Multivariable models were adjusted for age, sex, diabetes, smoking status, hypertension, hyperlipidemia, previous myocardial infarction, severity of coronary artery disease, and Killip class.

For statistical analysis, we used R version 4.4.3 (R Core Team, 2023) [26]. Missing data were imputed using the missRanger package [27], which employs a random forest approach to iteratively predict missing values. This method combines multiple random forest models to estimate missing values based on other observed data, enabling robust handling of both continuous and categorical variables. Missing data was assumed to be missing at random (MAR), meaning that the probability of missingness was related to observed data but not to unobserved values themselves. A two-sided p-value below 0.05 was considered statistically significant.

## Results

### COVID-19 trends

**Fig. 1:**
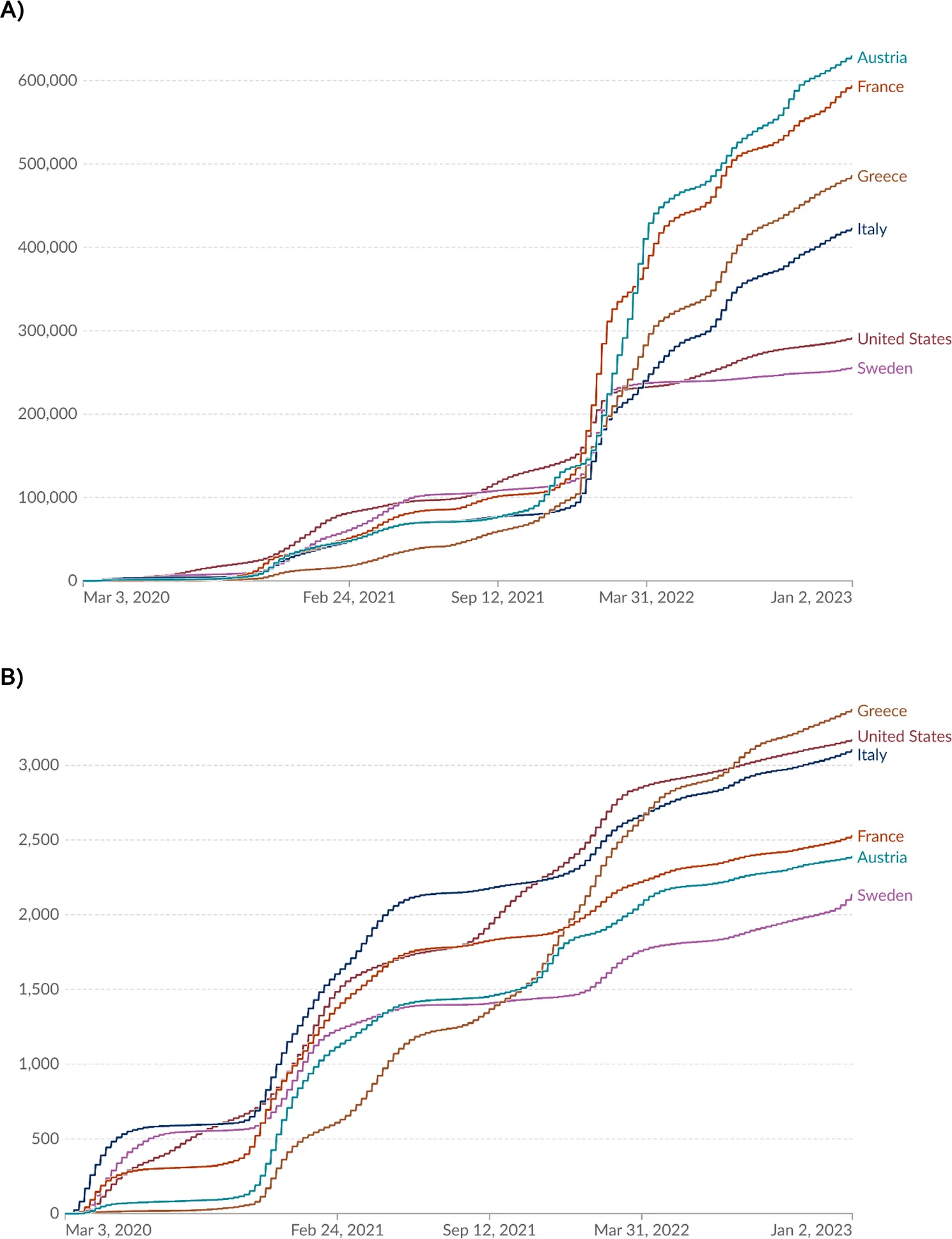
Cumulative incidence of new COVID-19 cases and mortality recorded from March 1 2020, to January 1, 2023. Cumulative incidence of confirmed COVID-19 cases and deaths per million population in Austria, France, Greece, Italy, Sweden, and the United States from March 1, 2020, to January 1, 2023. These graphs offer a comprehensive view of the COVID-19 pandemic's progression, highlighting differences in case and mortality trajectories among the six countries. **A** Cumulative incidence of confirmed COVID-19 cases per million population. The x-axis represents time, while the y-axis indicates the cumulative number of confirmed cases per million population. This figure illustrates the growth trajectory of COVID-19 cases, enabling direct comparisons of case accumulation rates across countries and providing insight into the impact of national pandemic responses. **B** Cumulative incidence of confirmed COVID-19 deaths per million population in Austria, France, Greece, Italy, Sweden, and the United States. The x-axis represents time, while the y-axis indicates the cumulative number of confirmed deaths per million population. This figure illustrates the variations in COVID-19 mortality trajectories, reflecting the pandemic's impact on population health and healthcare systems across different countries

An analysis of global COVID-19 data revealed significant variations in cumulative confirmed cases and deaths across specific countries. Figure 1A and B illustrate the trends in cases and mortality per million population. As shown in Fig. 1A, Austria reported the highest cumulative confirmed cases per million (629,374), followed by France (593,385) and Greece (485,751). Italy (422,563 cases per million) and Sweden (255,649 cases per million) had lower case burdens. The United States exhibited a moderate trend, with a significant but not the highest cumulative case burden. Figure 1B shows the cumulative number of confirmed COVID-19 deaths per million population. Greece exhibited the highest mortality rate among the selected countries, with 3,374 deaths per million, followed by Italy (3,102 deaths per million) and the United States (approximately 3,000 deaths per million). France (2,528 deaths per million) and Austria (2,388 deaths per million) showed moderately high mortality rates, while Sweden had the lowest cumulative mortality rate at 2,133 deaths per million. The other studies cover shorter periods between 2020 and 2021 when the trend in COVID-19 incidence is similar to Sweden’s.

### Patient characteristics

During the study period, we identified 3,180 patients hospitalized with TTS, with 69% (*n* = 2,189) of cases occurring before the pandemic and 31% (*n* = 991) during the pandemic. Females comprised 76% (*n* = 2,422) of the cohort, with a median age of 71. Detailed baseline characteristics are presented in Table 2. Notably, the median age of TTS patients increased slightly during the pandemic to 72 years, compared to 70 years in the pre-pandemic period. There was also a significant shift in the gender distribution, with females comprising 86% of cases during the pandemic, up from 71% before the pandemic.

**Table 2:** Baseline patient characteristics before the pandemic and during the pandemic period

Variable	Overall *N* = 3,180	Before COVID *N* = 2,189	COVID *N* = 991	*P*-value^2^
**Age** (years)	71 (62, 77)	70 (62, 76)	72 (63, 78)	< 0.001
**Sex**				< 0.001
Male	758 (24%)	624 (29%)	134 (14%)	
Female	2,422 (76%)	1,565 (71%)	857 (86%)	
**Smoking**				0.2
Never	1,433 (48%)	965 (47%)	468 (51%)	
Current	1,083 (37%)	758 (37%)	325 (35%)	
Former	440 (15%)	315 (15%)	125 (14%)	
**Diabetes**				0.14
No	2,698 (85%)	1,840 (85%)	858 (87%)	
Yes	466 (15%)	334 (15%)	132 (13%)	
**Hypertension**				0.4
No	1,368 (43%)	929 (43%)	439 (45%)	
Yes	1,781 (57%)	1,236 (57%)	545 (55%)	
**Previous Infarction**				< 0.001
No	2,770 (88%)	1,858 (86%)	912 (92%)	
Yes	373 (12%)	298 (14%)	75 (7.6%)	
**Previous PCI**				< 0.001
No	2,931 (92%)	1,982 (91%)	949 (96%)	
Yes	249 (7.8%)	207 (9.5%)	42 (4.2%)	
**Previous CABG**				0.001
No	3,094 (97%)	2,116 (97%)	978 (99%)	
Yes	86 (2.7%)	73 (3.3%)	13 (1.3%)	
**Indication for angiography**				< 0.001
Stable angina	135 (4.2%)	123 (5.6%)	12 (1.2%)	
ACS	2,413 (76%)	1,604 (73%)	809 (82%)	
Heart failure	202 (6.4%)	117 (5.3%)	85 (8.6%)	
Arrhythmia	34 (1.1%)	27 (1.2%)	7 (0.7%)	
Other	396 (12%)	318 (15%)	78 (7.9%)	
**Coronary artery disease**				< 0.001
Normal	2,425 (76.3)	1,565 (71.5)	860 (86.8)	
Single-vessel disease	372 (11.7)	282 (12.9)	90 (9.1)	
Multivessel disease	383 (12.0)	342 (15.6)	41 (4.1)	
**Killip class**				> 0.9
Class I	2,663 (91%)	1,844 (91%)	819 (91%)	
Class II	170 (5.8%)	119 (5.9%)	51 (5.7%)	
Class III	62 (2.1%)	42 (2.1%)	20 (2.2%)	
Class IV	36 (1.2%)	24 (1.2%)	12 (1.3%)	
**BMI** (kg/m^2^)	25.4 (22.6, 28.9)	25.5 (22.8, 28.8)	25.2 (22.1, 29.0)	0.3
**Height** (cm)	167 (162, 173)	167 (162, 174)	165 (160, 170)	< 0.001
**Weight** (kg)	71 (61, 83)	72 (62, 83)	70 (60, 81)	< 0.001
**Serum creatinine** (mmol/L)	71 (59, 85)	72 (61, 86)	67 (56, 81)	< 0.001

There was a significant difference in the distribution of coexistent coronary artery disease across the categories of normal, single-vessel, and multivessel disease (*p* < 0.001). 76.3% of patients had normal coronary arteries, with a higher prevalence during the pandemic (86.8%) compared to the pre-pandemic period (71.5%). Single-vessel disease was observed in 11.7% of patients, comprising 12.9% before the pandemic and 9.1% during the pandemic. Multivessel disease was identified in 12.0% of patients, with a higher prevalence before the pandemic (15.6%) and a markedly lower rate during the pandemic (4.1%). Comorbid conditions such as diabetes and hypertension showed no significant differences between the two periods. However, the incidence of previous myocardial infarction, prior PCI, and coronary artery bypass grafting (CABG) was significantly lower during the pandemic (9.5% vs. 4.2% for PCI and 3.3% vs. 1.3% for CABG). Additionally, anthropometric measures indicated that patients had slightly lower heights and weights during the pandemic, as well as reduced serum creatinine levels.

### In-hospital and discharge treatments

Table 3 presents the proportions of patients with TTS who received selected in-hospital interventions and discharge medications, stratified by the pre-pandemic and pandemic periods. Overall, the use of in-hospital treatments was similar across periods. Use of intravenous inotropes (4.0% pre-pandemic vs. 4.1% during the pandemic), IV diuretics (18.3% vs. 15.9%), IV nitrates (5.3% vs. 5.7%), and non-invasive ventilation (5.6% vs. 5.2%) did not differ significantly between the two groups. In contrast, several differences were observed in discharge medication patterns. The proportion of patients discharged with ACE inhibitors or ARBs was significantly lower during the pandemic (39.8%) than in the pre-pandemic period (46.6%; *p* = 0.012). Similarly, fewer patients were discharged on beta blockers during the pandemic (68.7% vs. 78.3%, *p* < 0.001), aspirin (62.4% vs. 68.2%, *p* = 0.023), and diuretics (16.0% vs. 21.6%, *p* = 0.011). No significant differences were observed in the use of statins, calcium channel blockers, or long-acting nitrates. For oral anticoagulants, apixaban use was notably higher during the pandemic (12.9% vs. 7.3%), whereas warfarin use was less frequent (1.1% vs. 5.3%). Among platelet inhibitors, use of ticagrelor declined from 22.8% to 13.5% during the pandemic, while clopidogrel use increased modestly (from 10.9% to 13.3%). These shifts were statistically significant (*p* < 0.001 for both classes).

**Table 3:** In-hospital and discharge treatments before and during the pandemic

Treatment	Before COVID *N* = 2,189	COVID *N* = 991	*P*-value
**In-hospital treatments (%)**			
IV inotropes	3.98	4.13	0.885
IV diuretics	18.26	15.87	0.242
IV nitrates	5.28	5.65	0.758
CPAP/BiPAP/NIV	5.64	5.22	0.732
**Discharge medications (%)**			
ACE inhibitors / ARBs	46.6	39.8	0.012
Beta-blockers	78.3	68.7	< 0.001
Statins	60.5	60.1	0.884
Aspirin	68.2	62.4	0.023
Diuretics	21.6	16.0	0.011
Calcium channel blockers	13.2	12.9	0.860
Nitrates (long acting)	5.9	5.4	0.714
Oral anticoagulants			< 0.001
*Warfarin*	5.3	1.1	
*Apixaban*	7.3	12.9	
*Other*	4.0	3.1	
Other platelet inhibitors			< 0.001
*Clopidogrel*	10.9	13.3	
*Ticagrelor*	22.8	13.5	
*Prasugrel*	0.07	0.23	

### Frequency of TTS, Killip class, and mortality

**Fig. 2:**
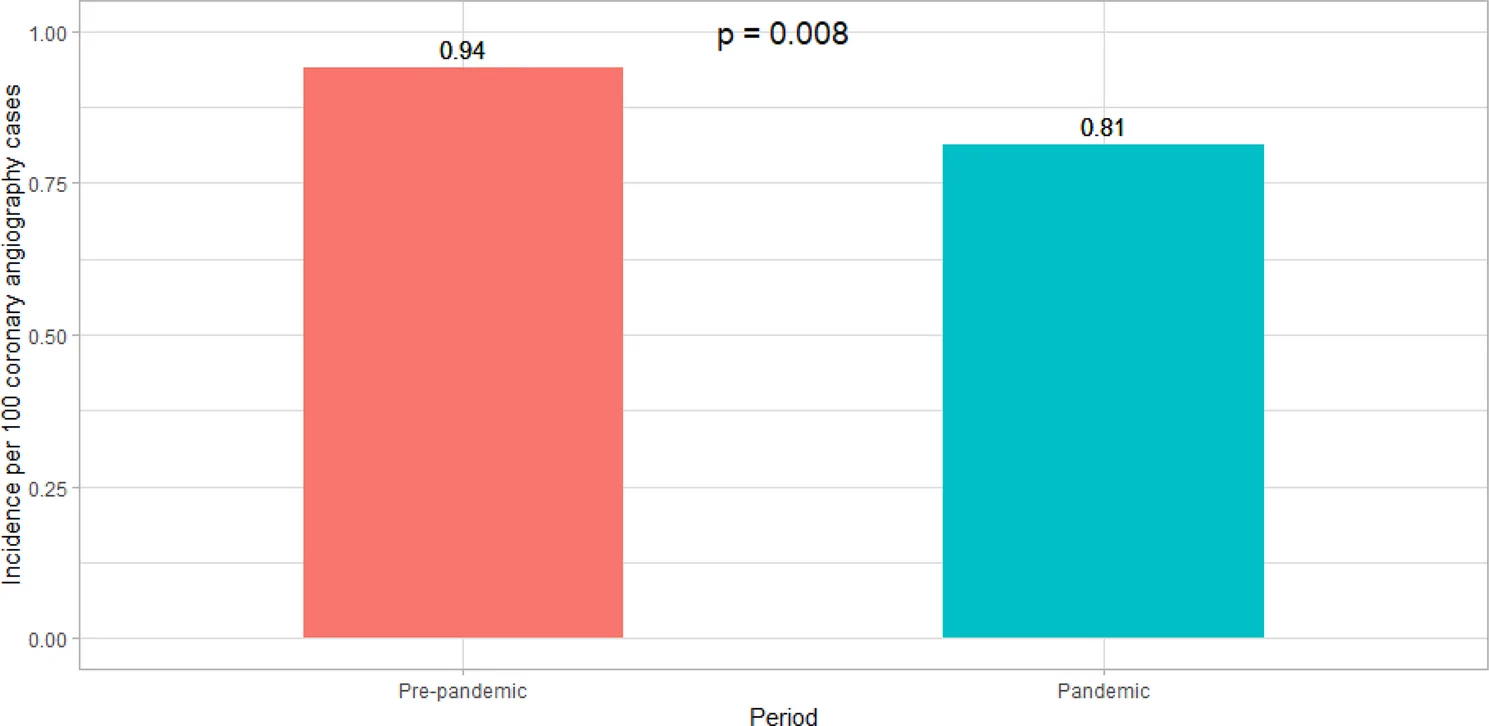
Bar chart illustrating the incidence of Takotsubo syndrome (TTS) per 100 coronary angiography cases in Sweden before and during the COVID-19 pandemic. This study aimed to determine the incidence and outcomes of TTS in Sweden before the pandemic (2015–March 2020) and during the pandemic (April 2020–December 2022). The x-axis represents the two time periods—pre-pandemic (left bar) and pandemic (right bar). The y-axis represents the incidence rate, calculated as the number of TTS cases divided by the total number of coronary angiography cases in each period, expressed as a percentage (i.e., multiplied by 100). Each bar displays the exact incidence rate above it, highlighting any changes in TTS incidence between the two periods

Figure 2 shows the incidence of TTS in Sweden during the study period. The incidence decreased from 0.94 per 100 coronary angiographies in the pre-pandemic period to 0.81 per 100 in the pandemic period. After adjustment for age and sex, the incidence of TTS was approximately 10% lower during the pandemic than during the pre-pandemic period (adjusted IRR, 0.90; 95% CI, 0.84–0.97; *p* = 0.008). To aid interpretation, Fig. 2 provides a visual summary of this finding. In plain terms, for every 100 patients undergoing coronary angiography, slightly fewer received a diagnosis of TTS during the pandemic than before it.

**Fig. 3:**
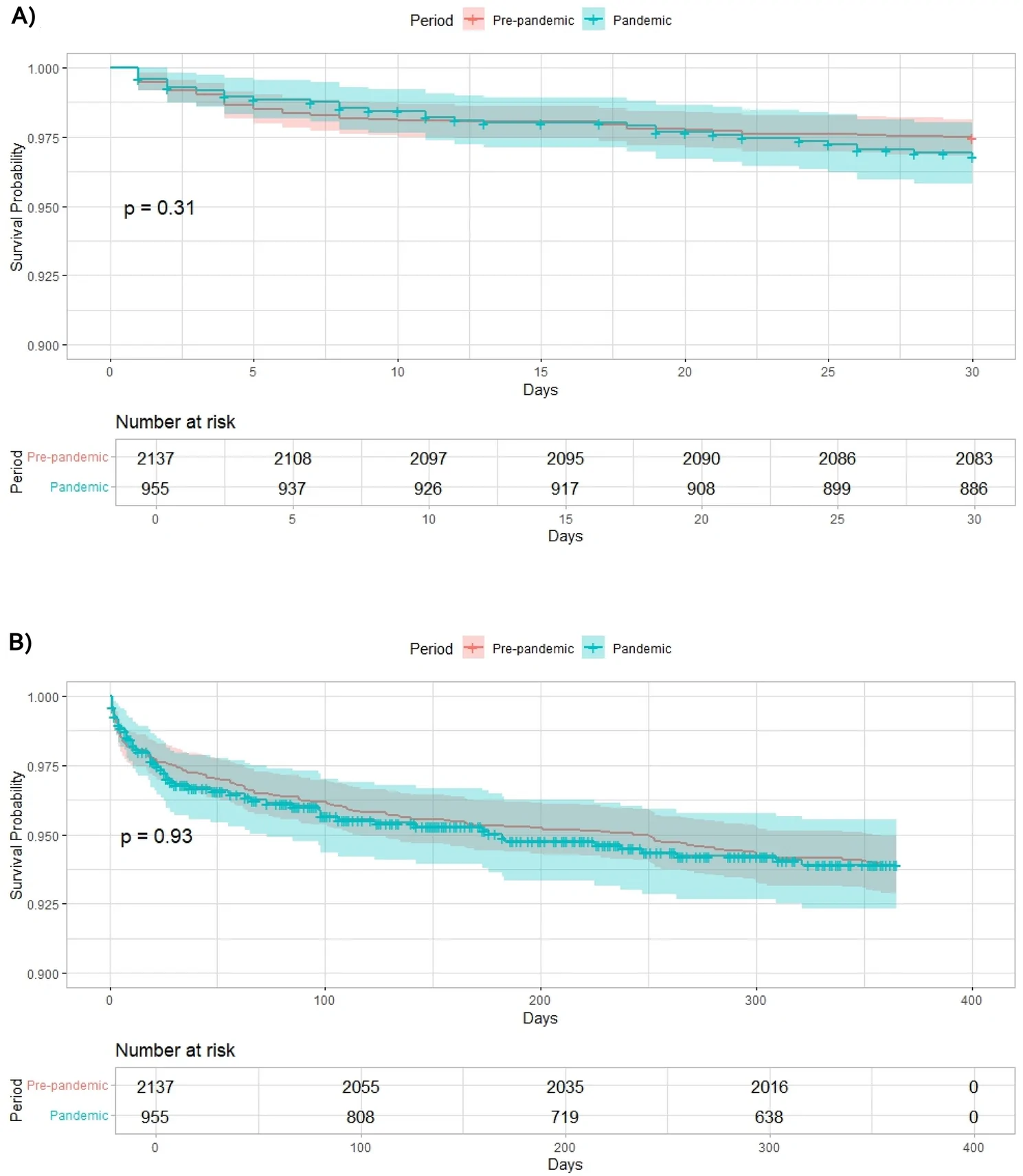
Kaplan-Meier Survival Curves Comparing Mortality in Takotsubo Syndrome Between Pre-Pandemic and Pandemic Periods. **A** 30-Day Kaplan-Meier Survival Curve for Takotsubo Syndrome: This plot illustrates the survival probability of patients diagnosed with Takotsubo syndrome over the first 30 days. The crude all-cause mortality rate for the cohort was 2.73% in 30 days, with 84 deaths. There was no significant difference in 30-day mortality between the pre-pandemic and pandemic periods (adjusted HR, 1.12; 95% CI, 0.69–1.78; P = 0.642). The shaded regions around the survival curves represent the 95% confidence intervals, and the "Number at Risk" table below shows the number of individuals still under observation at various time points over the 30-day period. **B** 1-Year Kaplan-Meier Survival Curve for Takotsubo Syndrome: This plot displays the probability of survival for the same cohort over a 1-year period. The crude all-cause mortality rate was 6.10% in 1 year, with 183 deaths. Similar to the 30-day findings, no significant difference in one-year mortality was observed between the two periods (adjusted HR 1.05, 95% CI 0.75–1.46, P = 0.780). The shaded regions represent the 95% confidence intervals, while the "Number at risk" table provides the count of patients still under observation at different time points up to 1 year

Most patients were classified as Killip Class 1, with similar proportions before (91.55%) and during (91.62%) the COVID-19 pandemic (Table 1). Killip Classes 2, 3, and 4 represented much smaller proportions, with no significant difference observed between the two periods (*P* = 0.974). Specifically, Killip Class 2 accounted for around 5%, Class 3 for approximately 2%, and Class 4 for just over 1% in both periods. The crude all-cause mortality rate for the cohort was 2.73% at 30 days, with 84 deaths, and 6.10% at 1 year, with 183 deaths. We found no difference in 30-day mortality (adjusted HR 1.12, 95% CI 0.69–1.78, *P* = 0.642; Fig. 3A) and one-year mortality (adjusted HR 1.05, 95% CI 0.75–1.46, *P* = 0.780; Fig. 3B) between the two periods among patients with TTS.

## Discussion

We analyzed data from 3,180 patients hospitalized with TTS, as recorded in the SCAAR registry, to evaluate the incidence and mortality of TTS during the COVID-19 pandemic. TTS decreased by 10% during the pandemic, while 30-day and one-year TTS-related mortality rates remained unchanged.

The COVID-19 pandemic caused significant increases in global economic, social, and psychological stress. Studies have shown a rise in mental health issues, including anxiety, depression, and post-traumatic stress, particularly among vulnerable groups such as women and healthcare workers [7–12, 28–31]. Global data indicate a substantial increase in depression and anxiety compared to pre-pandemic levels [28,29]. There is evidence that the pandemic had a disproportionately more negative effect on women’s mental health, potentially contributing to increased stress levels that are known triggers for TTS [30, 31]. Several studies have reported an increase in TTS incidence during the COVID-19 pandemic, with significant variations across countries and time periods. During the pandemic, a study from Italy’s Veneto region reported a notable increase in TTS cases, with an overall rise of approximately 15% in 2020 compared with previous years [15]. Another study in Greece also found a ~ 140% increase in TTS incidence during the pandemic period [16]. A study from Ohio, USA, reported a ~ 400% increase in TTS incidence during the pandemic [14]. Two other European studies reported no change in incidence during the pandemic. One is a single-center study from a tertiary hospital in France [18], and the other is a multicenter registry study from Austria [17].

The observed decrease in the incidence of TTS in Sweden during the COVID-19 period contrasted with the notable rise seen in the United States, Greece, and Italy, and there was no significant change in France and Austria. These diverging results suggest a potential link between national responses to the pandemic and health outcomes. Unlike other countries, Sweden adopted a more liberal approach with milder restrictions on social gatherings, providing recommendations to the public rather than imposing outright bans. The observed differences in TTS incidence may be attributed to varying levels of psychosocial stress across these nations, including differences in economic stability, public health responses, and social cohesion [32]. Sweden is known for its robust social security system and its commitment to egalitarian principles [33]. It consistently ranks among the world’s most equitable societies, offering widespread access to healthcare and social support mechanisms. Such a societal structure likely acts as a buffer against both acute and chronic stressors intensified by the pandemic, potentially reducing the occurrence of stress-induced affective disorders that may trigger TTS by providing economic security and social stability. Indeed, lower levels of depression, anxiety, and post-traumatic stress disorder have been reported in Sweden during the pandemic than in other countries [34, 35]. Conversely, the contrasting surge in TTS incidence in the US, Greece, and Italy may reflect the compounded effects of the pandemic on a population facing considerable disparities in healthcare access, economic inequality, and limited social support networks [36–40]. This highlights the need to consider broader sociocultural and economic factors when examining the epidemiology of stress-related cardiovascular diseases, especially during unprecedented global health crises such as the COVID-19 pandemic.

A trend towards lower incidence in Sweden was unexpected, as TTS incidence increased by 30% per calendar year from 2005 to 2013. A nationwide Swedish register study found a significantly reduced incidence of MI referred for invasive treatment strategy during the pandemic compared to pre-pandemic periods [41]. However, we have adjusted for this by normalizing TTS cases per 100 coronary angiography cases.

A recent study from Australia reported a 32% reduction in the incidence of ventricular arrhythmias requiring device therapy during the COVID-19 pandemic. This decrease coincided with measures of social isolation, potentially reflecting the impact of reduced psychosocial and physical stressors on arrhythmia burden [42]. This reduction in arrhythmias may reflect similar dynamics seen in Sweden’s reduced incidence of TTS. It is plausible that the societal and public health measures in Sweden, which focused on reducing isolation stress and maintaining a liberal approach to social activities, helped mitigate some of the psychosocial triggers of TTS.

Prior investigations utilizing the SCAAR registry in Sweden have demonstrated regional variations in the incidence of TTS among different counties in Sweden [4]. This variability may partially reflect disparities in resource availability, particularly concerning the capacity of specific centers to perform coronary angiography, leading to reliance on clinical diagnosis alone. Such limitations may have contributed to an underrepresentation of TTS cases during the COVID-19 pandemic. Additionally, it is likely that in some countries, an increasing number of TTS patients are undergoing coronary angiography due to heightened awareness of the disease and its clinical overlap with ACS [43]. During the pandemic, TTS patients exhibited a notable decline in the prevalence of prior myocardial infarction and PCI, as well as a decrease in the prevalence of the history of CABG.

On the other hand, there was a significant shift in clinical presentations, with an increase in cases presenting ACS and heart failure, and patients having less severe coronary artery disease. In contrast, the incidence of presentations of stable angina declined. These findings highlight TTS’s evolving clinical profile during the pandemic, likely influenced by shifts in patient behavior, heightened stress-related triggers, and altered healthcare accessibility.

A systematic review reported up to 23.3% in-hospital mortality among TTS patients during the pandemic, compared with 5.9% in the pre-pandemic period [44]. This increased mortality has been attributed mainly to TTS patients with concomitant severe pneumonia during COVID-19. However, our study lacks data on the presence or severity of pneumonia in TTS patients, which limits direct comparisons. Despite this, our findings indicate a stable TTS mortality rate during the pandemic. The absence of a difference in mortality aligns with our observed data on the Killip class.

Analysis of in-hospital and discharge treatments revealed notable changes in prescribing patterns during the pandemic. Specifically, fewer patients were discharged on ACE inhibitors/ARBs (39.8% vs. 46.6%) and beta-blockers (68.7% vs. 78.3%) during the pandemic. Early in the COVID-19 pandemic, concerns were raised regarding potential interactions between SARS-CoV-2 and the renin–angiotensin–aldosterone system, leading to uncertainty about the safety of ACEi and ARB therapy. Although major cardiovascular societies recommended continuation of these medications in the absence of specific contraindications, prescribing practices may have been influenced by this uncertainty during the initial phase of the pandemic. However, randomized clinical trials demonstrated that continuation of ACEi/ARB therapy was safe and was not associated with worse clinical outcomes in patients with COVID-19 [45, 46]. The lower use of these medications observed during the pandemic may reflect early changes in prescribing behavior, disruptions in healthcare delivery, and altered patient presentation patterns rather than evidence-based concerns regarding medication safety. A recent registry analysis suggested that RASi may benefit selected TTS subgroups with reduced left ventricular ejection fraction (LVEF) or diabetes [47]; however, we were unable to evaluate these subgroups due to a lack of LVEF and comorbidity-specific data. Importantly, in-hospital treatment patterns—including the use of intravenous inotropes, IV diuretics, and non-invasive ventilation—were similar between the two periods, consistent with our finding of no difference in clinical severity, as measured by Killip class. Despite reductions in certain discharge medications, no difference in short- or long-term mortality was observed, suggesting that these shifts in treatment practice did not negatively affect overall outcomes. Further research is needed to evaluate whether subgroup-specific effects may have been masked in our population-level analysis.

The strength of our study lies in its use of nationwide registry data, which provides near-complete coverage and ensures the dataset is highly representative of the entire population. This comprehensive approach minimizes selection bias and increases the generalizability of our findings to nations with similar pandemic strategies. Additionally, to the best of our knowledge, our study is the largest to date in terms of the number of TTS patients and related clinical events, comparing the pandemic and pre-pandemic periods, thereby providing robust statistical power to detect differences and evaluate outcomes effectively. Sweden’s more liberal approach to COVID-19 restrictions, combined with its strong healthcare and social support systems, could offer valuable insights for future healthcare planning during global crises.

There are some limitations to consider. First, because this is an observational study, causality cannot be firmly established, and unmeasured confounding factors may influence the results. This includes the potential impact of patients’ unwillingness to seek healthcare, as symptoms can resolve at home. Second, although the national registry ensures broad coverage, variations in data quality or completeness may still occur, particularly for specific subgroups or regions. However, all data are immediately registered online, thus limiting recall bias and missing values as these variables are compulsory to register. Third, the registry lacks post-discharge follow-up. However, the diagnosis of TTS was validated by another cohort [4]. Fourth, the generalizability of our study may be limited to nations with similar, more liberal strategies. Fifth, although our analyses did not reveal statistically significant differences in 30-day or 1-year mortality between the pre-pandemic and pandemic periods, the relatively wide confidence intervals indicate statistical uncertainty. These intervals encompass both potentially meaningful harm and benefit, which limits the precision of our estimates. Therefore, we cannot confidently rule out a pandemic-related effect on short- or long-term mortality among patients with TTS. More extensive studies or pooled analyses may help clarify these estimates in the future. Sixth, the SCAAR registry does not capture information on the emotional or physical triggers preceding TTS events, including COVID-19 infection or pneumonia. This lack of trigger data limits our ability to assess whether differences in stressor profiles—such as reduced psychosocial stress or increased physical illness during the pandemic—contributed to the observed changes in incidence or outcomes. A variable for active COVID-19 infection at the time of catheterization was introduced in SCAAR in March 2020. However, because rapid testing was unavailable during the early pandemic and the variable does not capture prior, recent, asymptomatic, or serologically confirmed infection, individual-level SARS-CoV-2 status was too incomplete to permit a reliable patient-level analysis. We were therefore unable to determine whether individual TTS cases were associated with concurrent SARS-CoV-2 infection, and the registry does not capture data on pneumonia or its severity. Finally, although the study was designed to compare the pre-pandemic and pandemic periods as distinct intervals, we did not stratify the pandemic period by specific COVID-19 waves. As the psychological and healthcare systems have evolved, this limits the ability to assess more granular temporal associations. Future analyses will address this by incorporating wave-specific stratification to better capture these dynamic influences.

The registry did not systematically capture subtype classification, such as apical, midventricular, basal, or focal variants, precluding assessment of whether the distribution of TTS phenotypes differed between the pre-pandemic and pandemic periods. Moreover, comparable information on TTS subtypes was not available in the studies included for comparison in the present work, limiting our ability to place potential subtype-specific differences in a broader context. Given that different TTS phenotypes may be associated with distinct clinical characteristics and triggers, future studies incorporating detailed imaging data may provide further insight into potential temporal changes in TTS presentation.

In conclusion, the incidence of TTS decreased compared to pre-pandemic levels, but mortality rates remained unchanged. Sweden’s lower incidence of TTS may partly reflect the country’s milder anti-pandemic strategies and more robust social security infrastructure than some other nations. 

## Supplementary information

Below is the link to the electronic supplementary material.

Supplementary material 1

## Data Availability

Data will be provided if requested by the editors.
