# Supplementary material for: Impact of the COVID-19 pandemic on the incidence and mortality of Takotsubo syndrome in Sweden, a report from the SWEDEHEART registry
Source: BMC Cardiovasc Disord. 2026 Oct 29;26:1018. doi: 10.1186/s12872-026-06889-0 (PMC13729366; doi:10.1186/s12872-026-06889-0)
Supplement: Supplementary file 1 [file 12872_2026_6889_MOESM1_ESM.docx]

University of Gothenburg

*Department of Molecular and Clinical Medicine*

SAHLGRENSKA ACADEMY AT UNIVERSITY OF GOTHENBURG

Elmir Omerovic, MD, PhD, Chair Professor of Cardiology

Xiulin Köhler PhD

Editor-in-Chief

BMC Cardiovascular Disorders

June 2026

Dear Editors,

Please find enclosed our revised manuscript entitled "Impact of the COVID-19 pandemic on the incidence and mortality of Takotsubo syndrome in Sweden — a report from the SWEDEHEART registry" by Gudmundsson and colleagues, submitted for reconsideration in BMC Cardiovascular Disorders.

We are grateful to the Editors and Reviewers for their careful and constructive assessment. In response, we have clarified whether individual-level SARS-CoV-2 infection-status data were available and addressed this explicitly in the Limitations; simplified the incidence analysis to a more transparent quasi-Poisson model and added a plain-language interpretation of the principal finding in the main text, with explicit reference to Figure 2 as a visual summary; expanded the Discussion of the observed reductions in ACE inhibitor/ARB and beta-blocker prescribing, including the subsequent randomized-trial evidence supporting the safety of renin–angiotensin system inhibition during COVID-19; clarified the ethics declaration to confirm compliance with national ethical regulations; and addressed the possibility of pandemic-related underdiagnosis and of differences in Takotsubo subtype distribution between periods. We have also incorporated our recent Nature Reviews Cardiology review of Takotsubo syndrome, providing readers with a comprehensive and up-to-date overview of its pathophysiology and clinical management.

We provide a detailed point-by-point response to each editorial and reviewer comment in the accompanying response letter.

We thank you for the opportunity to revise our manuscript and hope that the revised version is now suitable for publication in BMC Cardiovascular Disorders.

Sincerely,

Elmir Omerovic, MD, PhD, FESC, FAHA,

Chair Professor of Cardiology

| **Postadress** | **Postal address** | **Telephone:** |
| --- | --- | --- |
| Elmir Omerovic | Elmir Omerovic | Tel: +46 (0)31 342 2950 or |
| Wallenberg laboratoriet | Wallenberg Laboratory | Tel: +46 (0)70 456 0787 |
| Sahlgrenska Universitetssjukhuset | Sahlgrenska University Hospital | Fax: +46 (0)31 82 37 62 |
| 413 45 GÖTEBORG | SE-413 45 GÖTEBORG, Sweden | E-mail: elmir@wlab.gu.se |
